# Myocarditis with Cardiogenic Shock as the First Manifestation of Systemic Lupus Erythematosus

**DOI:** 10.5935/abc.20180216

**Published:** 2018-12

**Authors:** Jáder Buzati Rebelato, Caroline Ferreira da Silva Mazeto Pupo da Silveira, Tainá Fabri Carneiro Valadão, Fabrício Moreira Reis, Rodrigo Bazan, Silméia Garcia Zanati Bazan

**Affiliations:** Faculdade de Medicina de Botucatu - Universidade Estadual Paulista (UNESP), Botucatu, SP – Brazil

**Keywords:** Myocarditis, Shock, Cardiogenic, Lupus Erythematosus, System, Heart Failure, Echocardiography

## Introduction

Systemic lupus erythematosus (SLE) is a chronic inflammatory disease with multisystemic and autoimmune characteristics. It is the most common systemic autoimmune disease, occurring mainly in women between 20 and 40 years old, with a female-to-male ratio of 10:1. Even though the kidneys are classically considered the main organ affected by SLE, cardiomyopathy is one of the complications more frequently associated with morbidity and mortality in SLE patients.^1^ Cardiovascular impairment can be highly variable in terms of the affected structures and, in severe cases, may lead to cardiogenic shock.

In 2012, the Systemic Lupus International Collaborating Clinics (SLICC) published new criteria for SLE classification, aiming to optimize the diagnosis of cardiovascular impairment. However, cardiovascular disturbances are not part of the SLICC, even though there is such a high prevalence of cardiovascular disturbances in this population.^2^

## Case report

A 30-year-old Caucasian woman with a three-year history of arterial hypertension, who was an irregular user of captopril, sought medical attention due to a one-week history of dyspnea and chest pain. The patient presented with cold and clammy skin, dyspnea, hypotension, and tachycardia and was afebrile. A resting electrocardiogram (ECG) showed ST-segment elevation in all derivations. She was admitted for thrombolysis with streptokinase at the original hospital and was then transferred to the Tertiary Clinical Hospital. The patient was admitted to our emergency department on mechanic ventilation and was hemodynamically unstable and receiving norepinephrine.

A chest X-ray revealed cardiomegaly and pulmonary congestion; a transthoracic echocardiogram showed mild to moderate pericardial effusion, with diffuse hypokinesia of the left ventricle and significant systolic impairment with a left ventricular ejection fraction of 30%, as determined by the Teichholz method; the coronary angiography did not show any coronary lesions. Cardiac enzymes such as troponin and CKMB were elevated.

There was no recent history of infection. Additionally, blood cultures were negative three times, and serology for HIV was nonreactive.

The patient was diagnosed with myopericarditis, and hemodynamic support was provided with dobutamine, norepinephrine, and an intra-aortic balloon pump (IABP). Later, on the tenth day of hospitalization, the patient also showed signs of knee arthritis, altered consciousness and anisocoria.

**Figure 1:**
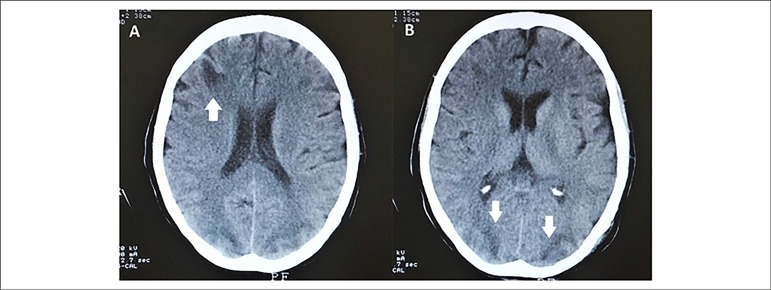
Computed tomography of the brain showing, in both A and B panels, hypodensity areas compatible with lacunar infarcts caused by vasculitis.

A computed tomography scan of the brain demonstrated multiple areas of cortical and subcortical hypodensity (Figure 1) and a brain arteriography showed a vasculitis pattern in the cerebral arteries. Antinuclear (ANA) and anti-DNA antibody tests were positive.

After the diagnosis of lupus myocarditis was made, on the twelfth day of hospitalization, the patient was started on immunosuppressive therapy with methylprednisolone (1 g intravenously once daily for three consecutive days) and later with cyclophosphamide (0.6 g/m^2^ intravenously once a month). There was significant clinical improvement, and a repeated transthoracic echocardiogram showed complete resolution of all changes. The patient remained asymptomatic, and on the twenty-eighth day was discharged from the hospital for outpatient clinical follow-up on 25 mg of captopril twice daily, 30 mg of diltiazem twice daily, 20 mg of omeprazole once daily, 70 mg of prednisone once daily and 250 mg of chloroquine once daily.

## Discussion

SLE is a chronic inflammatory multisystemic autoimmune disease with complex characteristics that affects mainly women, of which onset usually occurs between the ages of 16 and 55 years-old; it has a variable frequency in the general population, with an incidence of 1:200 in black women.^1^

Recently, the diagnostic criteria for SLE, collectively called the SLICC, have been revised and increased to a total of 17 criteria, from the 11 criteria of the previous 1997 classification.^2^

To diagnose SLE according to the new recommendations, four or more criteria must be met, and at least one must be clinical, whereas one must be immunological.^1^

In our patient, the diagnosis was confirmed due to the presence of serositis, neurological symptoms, and positive ANA and anti-DNA antibody testing (Table 1).

**Table 1:** Clinical and immunological criteria of the SLICC (Petri et al. 2012)^2^

CLINICAL CRITERIA	IMMUNOLOGICAL CRITERIA
1. Acute Cutaneous Lupus	1. ANA
2. Chronic Cutaneous Lupus	2. Anti-dsDNA
3. Oral ulcers	3. Anti-Sm
4. Nonscarring alopecia	4. Antiphospholipid Antibody
5. Synovitis involving >2 joints	5. Low Complement
6. Serositis	6. Direct Coombs Test
7. Renal manifestations	
8. Neurological Manifestations	
9. Hemolytic anemia	
10. Leukopenia/Lymphopenia	
11. Thrombocytopenia	

Although cardiovascular impairment is very common in patients with SLE, with a prevalence of up to 40-50% in postmortem studies, it is not part of the new diagnostic criteria; it is considered only associated damage due to long-term disease.^1-5^ It may manifest as pericarditis, myocarditis, Libman-Sacks endocarditis, pulmonary arterial hypertension or coronary artery disease; coronary artery disease is the most prevalent one, due to the inflammatory process of the disease itself together with the use of corticosteroids, which are commonly employed in the treatment of lupus.^6^

Due to the several impairment sites, the clinical manifestations may be quite variable and may range from asymptomatic or oligosymptomatic to cardiogenic shock, in the most severe cases of myocarditis.

In general, patients with lupus myocarditis are usually asymptomatic, with symptoms present in only approximately 5 to 10% of patients.^3^ However, severe heart failure may be the first manifestation of the disease.

Cardiogenic shock in lupus patients may have several etiologies, such as coronary artery disease, drug-induced cardiotoxicity (e.g., antimalarial drugs), pericarditis with cardiac tamponade, and valvular insufficiency secondary to valvular destruction, among other causes.^6^

A definitive diagnosis is made through anatomopathological analysis of an endomyocardial biopsy, which is not necessary in most cases. The endomyocardial biopsy has low sensitivity since the myocardial pattern may be focal in many situations.^5^ Thus, clinical suspicion combined with epidemiology, individual history and symptoms continue to be essential for diagnosis.

Inflammatory markers associated with the disease may be elevated in cases of myocarditis, along with reduction in serum complement levels. Among all the markers, the presence of anti-DNA antibodies has been associated with lupus myocarditis.^3^ An elevation in myocardial necrosis markers can occur; however, it is not related to clinical severity.^7,8^

The treatment of cardiogenic shock secondary to SLE begins with the same supportive treatment that is usually employed for patients with severe heart failure, regardless of the etiology.^2,4,5^ Thus, patients are usually started on inotropic drugs, vasodilators and vasopressors, and in patients who are refractory to the conventional clinical approach, mechanical support is required. The most common mechanical support, partly due to its availability, is an intra-aortic balloon pump; however, new devices for circulatory assistance may be used based on need.

Specific treatments for patients with severe left ventricular dysfunction associated with lupus myocarditis include high-dose corticosteroids; in some situations, such as in this patient, this involves pulse therapy with methylprednisolone, and other immunosuppressants (cyclophosphamide, azathioprine) or immunoglobuolins.^2,3,5,7^ However, the currently used treatments are not supported by scientific findings from controlled studies, due to the difficulty in performing such studies because of the rarity of this kind of presentation.

An early and precise diagnosis allows the implementation of an aggressive treatment of lupus myocarditis and leads to better outcomes, including the resolution of left ventricular systolic dysfunction, which may occur in up to 89% of cases within 6 months, according to reports in the literature^3^. However, an episode of lupus myocarditis seems to be a marker of worse prognosis in patients with systemic lupus erythematosus. In addition, perhaps cardiovascular manifestations should be included in future diagnostic criteria for SLE.
